# Evaluation of physical-mechanical properties, antibacterial effect, and cytotoxicity of temporary restorative materials

**DOI:** 10.1590/1678-7757-2017-0562

**Published:** 2018-08-20

**Authors:** Sonia Luque PERALTA, Sávio Bisinoto de LELES, André Lindemann DUTRA, Victoria Burmann da Silva GUIMARÃES, Evandro PIVA, Rafael Guerra LUND

**Affiliations:** 1 Universidade Federal de Pelotas, Faculdade de Odontologia, Departamento de Odontologia Restauradora, Programa de Pós-Graduação em Odontologia, Pelotas, RS, Brasil.; 2 Universidade Federal de Pelotas (UFPel) - Faculdade de Odontologia, Laboratório de Microbiologia Oral, Pelotas, RS, Brasil.

**Keywords:** Temporary dental restoration, Dental leakage, Solubility, Microbiology

## Abstract

The objective of this study was to compare selective physical-mechanical properties, antibacterial effects and cytotoxicity of seven temporary restorative materials (TRM): five resin-based materials [Bioplic (B), Fill Magic Tempo (FM), Fermit inlay (F), Luxatemp LC (L) and Revotek LC (R)], and zinc oxide-eugenol cement (IRM) and glass ionomer cement (GIC) as the controls.

**Material and methods:**

The physical-mechanical properties were evaluated by determining microleakage (ML), ultimate tensile strength (UTS) and Shore D hardness (SDH). In addition, the polymerization rate (Pr-1), depth of cure (DC), water sorption and solubility (WS/SL) were evaluated. The antimicrobial effects of the materials were assessed by biofilm accumulation of *Streptococcus mutans* (BT) and the direct contact test (DCT) by exposure to *Enterococcus faecalis* for 1 and 24 h, and cytotoxicity by MTT assay. The data were analyzed by ANOVA or Kruskall-Wallis tests, and a complementary post-hoc method (p<0.05).

**Results:**

Group B, followed by FM and GIC had significantly lower percentages of microleakage in comparison with the other groups; Groups FM and L showed the highest WS, while Groups R and FM showed the significantly lowest SL values (p<0.05). Group R showed the statistically highest UTS mean and the lowest DC mean among all groups. Group F showed the lowest *S. mutans* biofilm accumulation (p=0.023). Only the Group L showed continued effect against *E. faecalis* after 1 h and 24 h in DCT. The L showed statistically lower viability cell when compared to the other groups.

**Conclusions:**

These findings suggest the antibacterial effect of the temporary materials Fill Magic and Bioplic against *S. mutans*, while Luxatemp showed *in vitro* inhibition of *S. mutans* biofilm accumulation and *E. faecalis* growth. Regarding the cell viability test, Luxatemp was the most cytotoxic and Fill Magic was shown to be the least cytotoxic.

## Introduction

Temporary restorative materials are commonly used to seal the access cavity during the periods between visits and after the completion of endodontic therapy, their main function, both during and after the treatment, is sealing and preventing coronal microleakage.^1^ Despite the use of intracanal dressing between endodontic therapy appointments, some studies have reported the presence of residual intracanal microorganisms after this procedure.^2^ ^-^ ^4^ Temporary filling materials with good sealing ability and bactericidal properties may be advantageous to prevent bacterial invasions after an endodontic treatment. These materials can be divided into different groups according to their composition: reinforced zinc oxide–eugenol-based; calcium sulfate-based, resin-based composites; resin-modified glass-ionomer; and traditional glass-ionomer materials.^5^ Generally, all these materials are adequate if placed in a thickness of 3 mm or greater.^6^

Recently, new resin-based filling materials were introduced as temporary restorative materials.^6^ ^-^ ^7^ These materials contain monomers, initiator systems, fillers and additives. Resin-based temporary materials must be bonded to provide an effective seal, because they undergo polymerization shrinkage of 1 to 3%. ^8^ ^-^ ^11^ This contraction is compensated by the fact that they swell by absorbing water. These materials provide the best initial seal usually,^7^ but they lack antimicrobial properties.^8^

The antibacterial properties of restorative materials have been evaluated *in vitro* using various methods, and the agar diffusion test (ADT) was the standard assay in most of these studies, despite its limitations. Weiss, et al.^12^ (1996) introduced a direct contact test (DCT) that quantitatively measures the effect of direct and close contact between the test microorganism and the tested materials, regardless of the solubility and diffusivity of their components.^13^ ^-^ ^18^

The goal of this study was to investigate the physical-mechanical properties, antibacterial effects and cytotoxicity of seven different temporary fillings, as these may decrease the risk of caries development and failure of endodontic therapies.

## Materials and methods

**Figure 1:**
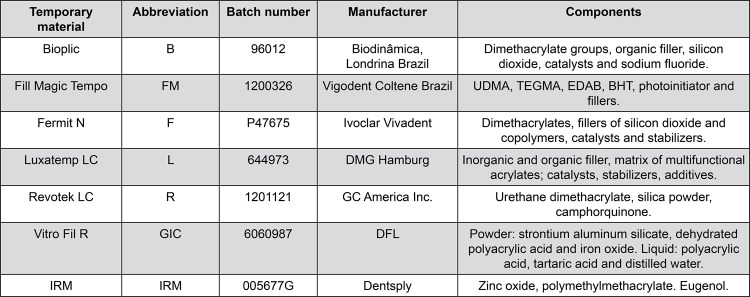
Composition of the materials used in this study

The materials tested in this study are described in Figure 1.

### Physical-mechanical properties

#### Microleakage (ML)

Ten recently extracted bovine incisors free of cracks were used. Cavities were prepared in the buccal surface of each tooth. Preparations were made by a single operator, using spherical diamond burs no. 1014 (KG Sorensen, Barueri, SP, Brazil) and no. 2082 (KG Sorensen, Barueri, SP, Brazil) fitted to a water-cooled high-speed handpiece. The saucer-shaped cavities were 3 mm in diameter and 1 mm deep, located in the middle-part of the buccal surface. The teeth were stored in distilled water and randomly divided into seven groups (n=10). After performing the preparations, the cavities were filled with the material, covered with polyester strips and polymerized for 20 s. The light-cured materials were light-activated with a LED light curing unit. The samples were immediately stored in blue methylene solution at 23°C for 24 h. Subsequently, they were submitted to thermal cycling for 500 cycles at temperatures ranging from 5° to 55°C, with a dwell time of 30 s. The root apexes of the teeth were sealed using chemically cured epoxy resin (Durepoxi^®^; Alba Química Indústria e Comércio Ltda., São Paulo, SP, Brazil). Two coats of nail varnish were applied on the tooth surfaces, except for the restoration, and a distance of 2 mm around their margins. The specimens were immersed in methylene blue solution at 23°C once more, for 10 min, and then washed in tap water for the same time and dried. The specimens were serially sectioned longitudinally in the buccal-lingual plane to obtain two (7 mm thick) slices that would be used to assess dye infiltration. Dye leakage was evaluated by two calibrated and blinded examiners using a stereomicroscope (Tecnival, Biosystems Ltda., Curitiba, PR, Brazil) at 40× magnification with an accuracy of 0.1 mm to measure the length of dye penetration in an image tool software. When the examiners disagreed regarding the dye leakage assessments, new examinations were done until a consensus decision was reached.

#### Water sorption and solubility (WS/SL)

Ten specimens of each material were made according to the standard ISO 4049:2009. Polymerized cylindrical specimens were produced in Teflon molds (diameter 15 mm, thickness 1 mm), dry-stored at 37°C and repeatedly weighed after 24 h intervals using an analytical digital balance (AUW220D; Shimadzu Corporation, Kyoto, Japan) accurate to 0.01 mg, until a constant mass was obtained. The specimens were then individually immersed in distilled water and stored at 37°C. After 7 days, the surface water of the specimens was removed and the mass of each specimen was recorded again. The specimens were dry-stored at 37°C and reweighed until reaching a constant mass. Water sorption and solubility were calculated as the percentage in mass gain or loss during the sorption and desorption cycles.

#### Kinetics of polymerization evaluated by RT-FTIR spectroscopy

The degree of conversion of the experimental materials was evaluated using Fourier transform infrared spectroscopy with a spectrometer (Prestige 21, Shimadzu, Ltd., Tokyo, Japan) equipped with an attenuated total reflectance device. The reflectance device was composed of a horizontal ZnSe crystal with a 45° mirror angle (PIKE Technologies, Madison, WI, USA). The IRSolution software package (Shimadzu, Columbia, MD, USA) was used in the monitoring scan mode using Happ–Genzel apodization in the range 1750–1550 cm^−1^ range, an 8 cm^−1^ resolution and 2.8 mm/s mirror speed. Using this configuration, one scan was acquired every 1 s during light activation. The degree of conversion was calculated as described in a previous study^19^ and based on the intensity of the carbon–carbon double-bond stretching vibrations (peak height) at 1635 cm^−1^, as well as using the symmetric ring stretching at 1610 m^−1^ from the polymerized and non-polymerized samples as an internal standard. The analyses were performed in triplicate (n=3). The data were plotted and curve fitting was applied using logistic non-linear regression. In addition, the polymerization rate (*Pr* ^*-1*^) was calculated as the degree of conversion at time t subtracted from the degree of conversion at time t − 1. The coefficient of determination was greater than 0.98 for all curves.

#### Depth of cure (DC)

Depth of cure was analyzed by the scraping method. The materials were put into a cylindrical silicone mold (6 mm diameter, 20 mm height) and irradiated through a polyester strip for 20 s. The material was extracted from the mold and the uncured material (if any) was removed. The maximum thickness of the cured material was measured with a digital caliper (n=3).

#### Ultimate tensile strength (UTS)

Ten dumbbell-shaped specimens (length 10 mm x width 5 mm x constriction 1 mm) were prepared for each group using elastomer molds. The top and bottom surfaces were light-activated for 20 s. After fabrication, the tensile test was conducted in a mechanical testing machine (DL500; EMIC, São José dos Pinhais, PR, Brazil) at a crosshead speed of 1mm/min until failure. UTS values were calculated in MPa.

#### Hardness measurements

The measurements were made in accordance with ASTM D2240 using the Shore D hardness (SDH) scale tester (PanTec; Panambra Ind. e Técnica SA, São Paulo, SP, Brazil). The measurements were made on specimens approximately 1.5 mm thick. Five specimens *per* group were tested and four readouts were taken at four different positions on each specimen. Mean and standard deviation were calculated from all readouts.

## Microbiology test

### Biofilm accumulation test

*Streptococcus mutans* UA159 is one of the major bacterial species responsible for dental caries,^20^ it was cultured overnight in brain heart infusion broth (BHI) at 37°C in an anaerobic atmosphere. The bacterial suspension obtained was adjusted to an optical density (OD) of 0.5 at 600 nm.

Specimens measuring 6mm in diameter and 1mm thick were suspended into the cavities of a 24-well plate. An aliquot of 2 mL of ultrafiltered tryptone-yeast extract broth (UTYEB) supplemented with 1% sucrose and 20 μL of bacterial suspension were inoculated into each well. The biofilms on discs were washed 3 times daily in 0.9% NaCl and transferred to a new plate with fresh UTYEB containing 1% sucrose for 24h. This procedure was repeated for 3 days. All plates were incubated at 37°C in an environment of 5-10% CO_2_ (Anaerobac; Probac do Brasil Produtos Bacteriológicos Ltda., Santa Cecília, SP, Brazil) in anaerobic jars (Probac do Brasil Produtos Bacteriológicos Ltda., Santa Cecília, SP, Brazil). After 72 h of biofilm growth, the discs containing the biofilms were washed 3 times in 0.9% NaCl and individually transferred to microcentrifuge tubes containing 1 ml of 0.9% NaCl. The tubes were sonicated at 30 W for 30 s (Sonicator DE S500, R2D091109, Brazil) to detach the biofilms formed on the discs.

To determine bacterial viability, an aliquot of 100 μl of the biofilm suspension was serially diluted in 0.9% NaCl up to 10-7 and 2 drops of 20 μl of each dilution were inoculated on BHI agar (Difco Becton Dickinson, Sparks, USA) to determine the number of viable microorganisms. ^21^ The plates were incubated at 37°C for 72 h in an environment of 5-10% CO_2_ (Anaerobac; Probac do Brasil Produtos Bacteriológicos Ltda., Santa Cecília, SP, Brazil) produced in anaerobic jars (Probac do Brasil Produtos Bacteriológicos Ltda, Santa Cecília, SP, Brazil). Colony forming units (CFU) were counted and the results were expressed in CFU/mg of biofilm dry weight.^22^

### Modified Contact direct test

*Enterococcus faecalis* (ATCC 4083) isolated from a periapical abscess was used as the microorganism tested, it was cultured overnight at 37°C in tryptic soy agar (TSA) plates in an aerobic atmosphere. *E. faecalis* was inoculated in tryptic soy broth (TSB) and the bacterial turbidity was adjusted to an optical density of 0.5 at 600 nm.

Cylinders measuring 6 mm in diameter and 1mm thick were placed in a 96-well plate. Subsequently, 10 µL of bacterial suspension was placed on the surface of the materials tested. Strain suspensions (10 µL) placed in uncoated wells served as non-exposed (positive) controls. Materials incubated without bacteria served as negative controls. All samples were incubated aerobically at 37°C for 1 and 24 h, in >95% humidity; then 240 mL of TSB was added to each of the wells and gently mixed using a pipette for 1 min. Serial dilutions were prepared in TSB; plated onto TSA and incubated in an aerobic environment at 37°C for 24 h. The CFU were counted and CFU/mL was calculated.^23^ The experiments were performed in duplicate.

## Cytotoxicity assay

Discs of each material were fabricated under aseptic conditions in sterile cylindrical silicone discs measuring 5 mm in diameter and 1 mm high. The cytotoxicity of the materials was assessed after 24 h. The extraction was made in cell culture medium and after the extraction, the vials were incubated at 37°C for 24 h. Control samples containing only culture medium were treated similarly and undiluted extracts were used for testing.

The viability of fibroblast (NCTC clone 929) cells was determined by measuring the reduction of cellular 3-(4,5-dimethylthiazol-2-yl)-2, 5-diphenyltetrazolium bromide (MTT – Sigma, St. Louis, MO, USA) into water-insoluble formazan. Briefly, the cells were seeded at a density of 2x10^4^ cells per well in a volume of 200 μl in 96-well plates and grown at 37°C in a humidified atmosphere of 5% CO_2_ 95%. After incubation for 24 h, the medium was aspirated from all wells and replaced with 200 μL/well extract or control medium and incubated for 24 h. Then, the incubation medium was removed, and subsequently 180 ml of medium and 20 ml MTT (5 mg MTT/ml solution) were added to each well. The plates were incubated for an additional 4 h and the medium was discarded. Dimethyl sulfoxide (DMSO) was added to each well and the formazan was solubilized on a shaker for 5 min. The formazan content of each well was computed as a percentage of the control group (untreated cells). All assays were repeated 3 times to ensure reproducibility. Cytotoxicity responses were rated as severe (30%), moderate (30%–60%), slight (60%–90%), or noncytotoxic (>90%).^24^

## Statistical analyses

The equality of the variances and the normal distribution of the errors were checked for all tested response variables. Those that did not met these conditions were submitted to transformations attempting to fulfill parametric assumptions.

The following data: microleakage; water sorption; depth of cure, and direct contact test for 1h, were submitted to non-parametric Kruskal-Wallis test (p<0.05). Microbiological assays: CFU count data were non-normal and log transformed. Subsequently, statistical analyses were performed using the transformed data. A log_10_ transformation of each CFU count was performed to normalize the data before the statistical evaluation due to the high range of bacterial numbers. Then, to determine viable bacteria counts, statistical analyses were performed using one-way ANOVA and the Fisher’s least significant difference (LSD) *post hoc* test for pairwise comparisons among means. For UTB: solubility; Shore D hardness; direct contact test for 24 h and biofilm accumulation, ANOVA and a complementary Student-Newman-Keuls tests were used. All statistical tests were performed using the program Sigma Stat^®^ for Windows Software^®^, Version 3.5 (Systat Software, Inc., Point Richmond, CA, USA), using a preset alpha of 0.05.

## Results

### Physical-mechanical properties

**Figure 2:**
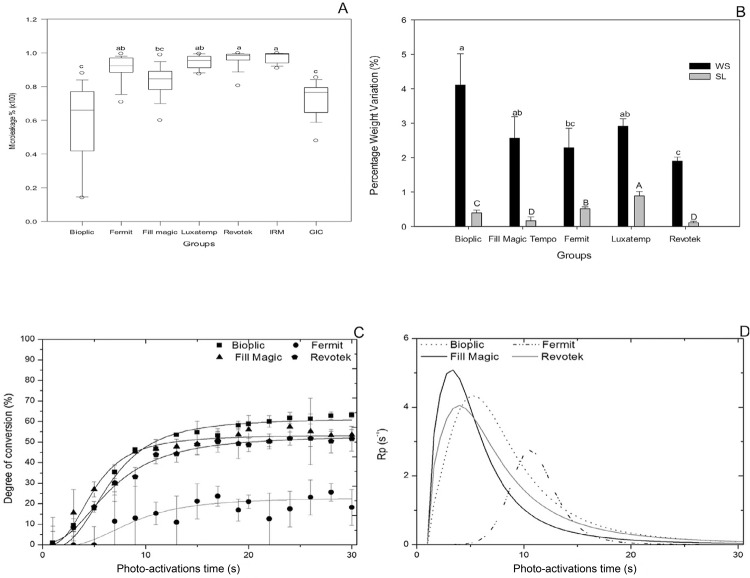
(A) Microleakage after 1000 cycles, (B) water sorption and solubility expressed in %wt, (C) Degree of conversion and (D) Polymerization rate Pr-1 of materials tested. Different letters represent statistically significant differences between groups (p<0.05)

Regarding microleakage, the cavities restored with materials B, FM and GIC showed significantly lower leakage than the other groups (Figure 2A). For the groups of materials, the following mean percentages of microleakage were found: IRM=97.2%; R=96.0%; L=94.2%; F=90.1%; FM=83.2%; GIC=72.3%; and B=58.8%. Statistically, significant differences were found when comparing R, IRM, B and GIC with the other groups.

Water sorption and solubility results are shown in Figure 2B. The highest sorption values were obtained for groups B, FM and L, followed by R and F, which showed the lowest sorption (p<0.001). The highest solubility values were found in group L, and the lowest levels in groups FM and R (p<0.001).

Figures 2C and 2D show the degree of conversion (DC%) and development of the polymerization rate (*Pr*) as a function of time for the different materials tested. Group F presented a low final DC and Pr values lower than 30%.

The depth of cure showed statistically significant difference (p<0.001). Group R showed a lower value than group B, which presented the highest value. The other groups presented intermediate values (Table 1).

**Table 1:** Mechanical properties of the different materials tested (Mean ± SD)

Group	Ultimate Tensile Strength (Mpa)	Depth of Cure (mm)	Shore D Hardness
Bioplic	8.98±2.4^b^	8.50^a^	55.13±1.8^b^
Fill Magic tempo	5.78±0.9^c^	7.31^b^	54.56±2.8^b^
Fermit	9.58±1.9^b^	7.57^b^	56.00±2.8^b^
Luxatemp	4.43±1.4^c^	6.71^b^	32.01±1.9^c^
Revotek	32.8±3.2^a^	5.55^c^	77.50±1.8^a^

Different lower case letters in the columns represent statistically significant differences between groups (p<0.05)

Regarding ultimate tensile strength (Table 1), group R showed higher statistically significant difference than the other materials. Groups FM and L presented the lowest value, and intermediate values were observed for groups B and F (p<0.001).

Shore D hardness results are shown in Table 1. Group R presented a statistically higher value than L. The other materials B, FM and F showed statistically similar values (p<0.001).

### Microbiological effect

**Figure 3:**
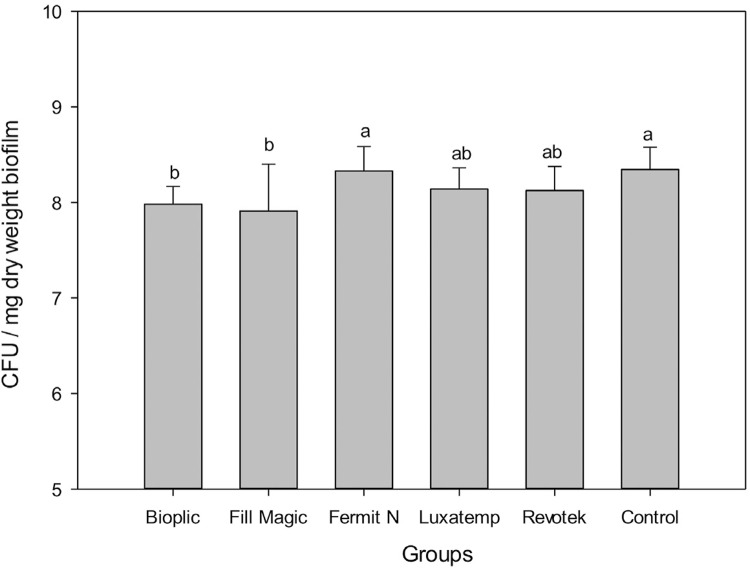
*S. mutans* (UA159) accumulation test after 3 days of biofilm formation under continuous exposure to 1% sucrose. Different letters represent statistically significant differences between groups (p<0.05)

The development of *S. mutans* in biofilm was significantly affected by the materials (Figure 3), except for group F (p=0.023). The results of the DCT test after 1 h showed that groups L and B were significantly more potent bacterial growth inhibitors than the other materials (p<0.001).

**Figure 4:**
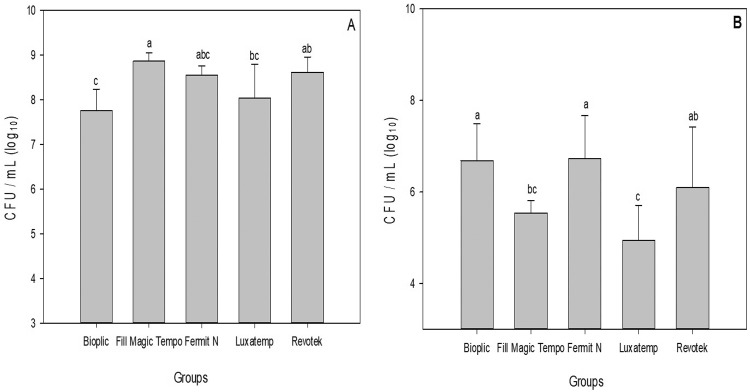
Survival de *E. faecalis* (ATCC4083) after direct contact with temporary filling materials; (A) temporary filling for 1 h. (B) temporary filling for 24 h. All materials were light-cured. Different letters represent statistically significant differences between groups (p<0.05)

Furthermore, the DCT (test) results after 24 h (Figure 4B) showed that groups B, F, and R presented higher CFU values than FM and L (p<0.001).

Luxatemp continued to be effective for 1 h and 24 h after the DCT against *E. faecalis*. Fill Magic Tempo showed antimicrobial activity only after 24 h of the DCT.

### Cytotoxicity assay

**Figure 5:**
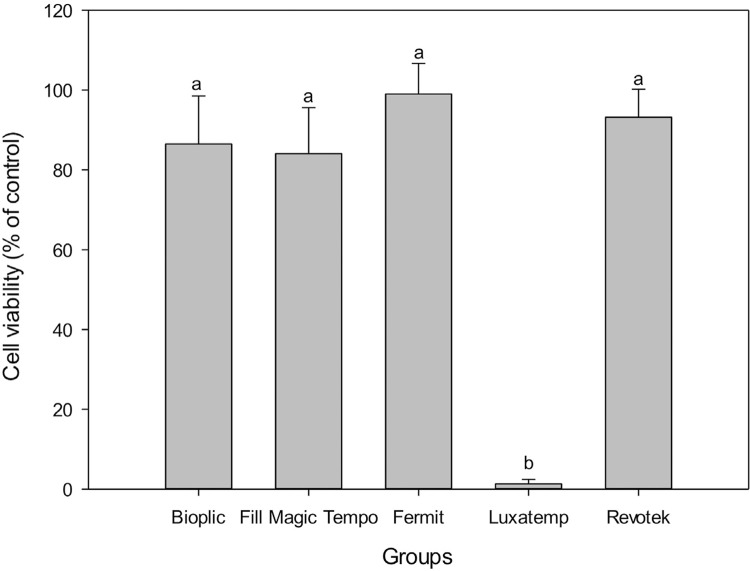
The cytotoxic effects after exposure to MTFs in L929 fibroblast cells. Results are expressed as mean and standard deviation. Different letters indicate statistically significant differences (p<0.05)

Luxatemp was statistically more cytotoxic that all other materials tested (p<0.001). Whereas, Fermit showed cell viability close to 100%. Cytotoxicity data are summarized in Figure 5.

## Discussion

*In vitro* tests remain an indispensable method for initial screenings of dental materials and setting a theoretical maximal amount of leakage that could be present *in vivo*.^25^ Dye leakage is the cheapest and fastest method to test the sealing ability of restorative materials.^26^ However, there are drawbacks associated with this test. In addition to the difficulties of reproducing all challenges faced in the oral environment, the wide methodological variation in microleakage tests found in the literature makes a reliable comparison between studies difficult.^27^ Some examples of factors leading to these difficulties are the different microleakage measurement methods; dyes and markers used with different composition; pH; molecular weight; and concentration.^25^ ^,^ ^28^ Moreover, different immersion times have been reported, ranging from 4 h to 72 h.^26^ In this study, immersion in methylene blue solution 0.5% for 24 h was adopted. These parameters were reported in a systematic review about microleakage tests as the most commonly found parameters in the studies evaluated.^27^

Microleakage through temporary filling material is a very important property regarding its ability to seal the cavity, preventing contamination of the canal that could lead to treatment failure. Furthermore, dentin is a heterogeneous, less mineralized and moist tissue, making the bonding process a challenge and affecting the durability of the bond and the marginal sealing of restorations. In this study, better marginal sealing was observed using GIC, FM and B. The findings of this study for the material B corroborate those observed by Pieper, et al.^7^ (2009). These results can be explained because the materials are resin-based, thus, requiring a light source for their polymerization that produced contraction; and in the presence of water there was expansion. ^8^ The result obtained for the group GIC differed from those of other studies.^7^ ^,^ ^13^ Pieper, et al.^7^ (2009) found higher microleakage in GIC restorations than in those performed with B. However, the results from this study for GIC were similar to those published by Castro, et al.^14^ (2013), despite the difference in scale and methodology used in both studies. GIC presented little to no exothermic reaction or shrinkage during setting, having no free monomer in the set matrix, and bonding to the tooth structure. Based on its bonding potential, it could be expected that the marginal sealing produced by GICs would be good.^5^ ^,^ ^15^ Different studies have indicated that IRM showed higher microleakage values,^15^ and this study found similar results.

WS results could be associated with microleakage. In this study, group B showed better sealing and higher WS values (Figures 2A and 2B). Group L also had a good sorption but also showed higher solubility, which facilitates microleakage. Group R showed lower WS, probably because no expansion gaps that facilitate microleakage appeared. Pieper, et al.^7^ (2009) evaluated the WS and SL of the material B used in this study, however, it was not possible to make a comparison because they used a different unit. The sorption and solubility properties of resinous materials are affected by many factors, like: chemical composition; presence of hydrophilic constituents in the resin matrix;^17^ and structural parameters of the polymeric network, such as cross-linking density and porosity.^18^

Regarding polymerization kinetics, group L was not considered in the results because it was not possible identify a peak (1610 cm^−1^) for analysis. The polymerization rate of the other materials was evaluated and kept stable after 20 s of light polymerization. In Figure 2D, the highest rate of polymerization can be observed to occur in the first 5 s, probably indicating that more than one co-initiator may be used in their composition.^29^

These materials have advantages, such as polymerization occurring in an increment exceeding 4 mm. This property was evaluated by the depth of cure, and we must highlight that in all materials, more than 4 mm was polymerized, except for group R. The common characteristic of these materials is their translucency, which facilitates the passage of light, possibly indicating a smaller amount of inorganic filler. Group R showed the lowest depth of cure, this can be explained by its inorganic composition. This material is used for provisional prosthetic restorations and for this reason it must have good mechanical resistance.

This is the first research on the antimicrobial effect of temporary filling materials against *S. mutans* biofilm formation. *S. mutans* are known to be important in the development of caries^30^ and that biofilms are more resistant than planktonic microorganisms. Groups B and FM showed better effect against *S. mutans*, however, group FM was the only material to present antibacterial properties when in contact with S. mutans for 3 days and over 24 h in the DCT with *E. faecalis.*

We chose *Enterococcus faecalis* to evaluate the antibacterial effect in this study because it is the most commonly found bacteria after unsuccessful endodontic treatments.^31^ Several *in vitro* methodologies have been used to evaluate the antibacterial properties of restorative materials . The agar diffusion test (ADT) was the standard assay in most studies.^23^ The direct contact test (DCT) is a reproducible method that simulates the contact of the tested microorganisms with the material. This test provides information on bacterial viability and growth rate,^32^ by allowing the number of the viable bacteria to be estimated after incubation periods in direct contact with the material. In endodontics, the difficulties to eliminate *E. faecalis* from the root canal system may be related to its ability to penetrate into dentinal tubules and organize itself into biofilms. The antibacterial activity of materials may help to eliminate microorganisms present in the root canal, thus, improving the success of endodontic treatments.

After 24 h of DCT, group L showed the strongest antibacterial activity against *E. faecalis*, followed by group FM. The lowest antimicrobial activity among all samples was from groups R and F. The materials R and F were also assessed in a previous study regarding their antibacterial activity.^2^ Similarly, to our findings, material R revealed no antimicrobial effect against *E. faecalis;* whereas, the results of material F differed from those found in this study, because in the referred study, F also showed no antibacterial effect.^3^ After 24 h, materials FM and L showed antimicrobial activity against *E. faecalis*.

Group L showed the highest cytotoxicity, since there are many components that influence biocompatibility, such as: monomers; photo-initiators; or fillers.^33^ ^,^ ^34^ Manufacturers are usually reluctant to reveal complete information about their products, however, the cytotoxic effect of Luxatemp could explain the antibacterial effect against *E. faecalis* after 24 h in the DCT. Cytotoxicity is preferred as a pilot project test and an important indicator for toxicity evaluation of biomaterials as it is simple, fast, has a high sensitivity and can save animals from toxicity. The cytotoxicity test is one of the most important indicators of the biological evaluation system *in vitro*, and considering the ongoing progress of modern cell biology, experimental methods to evaluate cytotoxicity are also continuously being developed and improved.^35^

Based on the results of this study, we can conclude that Bioplic and Fill Magic showed lower microleakage, Revotek and Fill Magic presented lower solubility and the highest polymerization rate occurred in the first 10 seconds, except for Fermit. Regarding the *S. mutans* biofilm accumulation assay and direct contact test with *E. faecalis*, Fill Magic tempo showed the strongest antibacterial effect. Regarding the cell viability test, Luxatemp was the most cytotoxic, whereas Fermit demonstrated higher cell viability.
